# Spatial distribution and determinants of iron supplementation among pregnant women in Ethiopia: a spatial and multilevel analysis

**DOI:** 10.1186/s13690-021-00669-2

**Published:** 2021-08-10

**Authors:** Chilot Desta Agegnehu, Getayeneh Antehunegn Tesema, Achamyeleh Birhanu Teshale, Adugnaw Zeleke Alem, Yigizie Yeshaw, Sewnet Adem Kebede, Alemneh Mekuriaw Liyew

**Affiliations:** 1 School of Nursing, College of Medicine and Health Sciences and Comprehensive specialized hospital, University of Gondar, Gondar, Ethiopia; 2 Department of Epidemiology and Biostatistics, Institute of Public Health, College of Medicine and Health Sciences, University of Gondar, Gondar, Ethiopia; 3 Department of Physiology, School of Medicine, College of Medicine and Health Sciences, University of Gondar, Gondar, Ethiopia

**Keywords:** Iron supplementation, Spatial analysis, Multilevel analysis, Individual level, Community level, Ethiopia

## Abstract

**Background:**

Maternal anemia continues as a global public health concern particularly in developing countries including Ethiopia. It is associated with an increased risk of maternal death, obstetric complications, preterm birth, and low birth weight. Even though maternal anemia is the commonest problem in Ethiopia, there is limited evidence on the spatial distribution and determinants of iron supplementation. Therefore, this study aimed to investigate the spatial distribution and determinants of iron supplementation among pregnant women in Ethiopia.

**Method:**

A secondary data analysis was conducted based on the 2016 Ethiopian Demographic and Health Survey (EDHS) data. A total weighted sample of 7589 women was included for analysis. For the spatial analysis; ArcGIS version 10.6, and SaT Scan version 9.6 statistical software were employed to explore the spatial distribution, and to identify significant hotspot areas of iron supplementation in Ethiopia. For the determinant factors, multilevel logistic regression analysis was fitted to identify significant individual and community level determinants of iron supplementation. Deviance, Median Odds Ratio (MOR), and Intra-class Correlation Coefficient (ICC) were used for model comparison and for assessing model fitness. Variables with a *p*-value of less than 0.2 in the bivariable analysis were considered in the multivariable multilevel analysis. In the multivariable multilevel analysis, the Adjusted Odds Ratio (AOR) with 95% Confidence Interval (CI) was used to declare significant determinants of iron supplementation.

**Results:**

The spatial distribution of iron supplementation was significantly varied across the country with Global Moran’s index value of 0.3 (*p* < 0.001). The SaTScan analysis identified a total of 271 significant clusters, of these 89 clusters were primary clusters located in the Southwest Somali and Central Oromia regions (LLR = 66.69, *P* < 0.001, RR = 1.35). ANC visit (AOR = 3.66, 95%CI: 3.21, 417), community education [AOR = 1.31, 95%CI, 1.07, 1.59), media exposure (AOR = 1.33, 95%CI: 1.15, 1.53), distance to health facility (AOR = 1.32, 95%CI: 1.16, 1.50), region and household wealth index were statistically significant determinant factors of iron supplementation.

**Conclusion:**

Iron supplementation among pregnant women were significantly varied across the country. Therefore, the finding of this study could help to design effective public health interventions targeting areas with low iron supplementation and maternal health services should be delivered in all areas of our country. Besides, public health programs should enhance iron supplementation through promoting ANC visits, media exposure, and giving special emphasis to marginalized and remote areas.

## Background

Globally, the prevalence of anemia among pregnant women is estimated to be 41.8%. Of which half of this burden occurs due to iron deficiency or lack of iron tablet supplementation [1, 2]. Iron Deficiency Anemia (IDA) is one of the foremost frequently observed preventable types of anemia among pregnant women in developing countries [2, 3]. The demand for iron is an increase for pregnant women due to loss of blood following delivery or increase demand of iron to the fetus [4]. For pregnant women, the recommended iron supplementation is 20 mg per day which is higher than the requirement for non-pregnant women [5]. This is because the quantity of iron absorbed from the diet is not sufficient during pregnancy and infancy [6]. Consistent with the advice of the World Health Organization (WHO), all pregnant women regardless of their hemoglobin level status should get iron supplementation [7].

Anemia during pregnancy increases the risk of maternal death, obstetric complications, preterm birth, and low birth weight [8–10]. Timely supplementation of the recommended dosage prevents the after-mentioned complications of maternal and child health [11, 12].

Factors associated with iron supplementation among pregnant women include advanced maternal age [13, 14], parity [14], knowledge of about anemia and diagnosis of anemia [14, 15], and use of other food supplements [16]. The coverage of iron supplementation among pregnant women is low in Ethiopia, below the recommendation of WHO [7]. Besides, it varies from region to region. So, exploring the spatial distribution among pregnant women is very important for policymakers to take targeted intervention by identifying the riskiest areas for the problem. This targeted intervention would help to maximize the optimal utilization of scarce resources. In addition handling different determinants of iron supplementation among pregnant women also good strategy to reduce the burden of anemia and improve the quality of care among pregnant women. Therefore, the aim of this study was to explore spatial distribution and associated factors of iron supplication among pregnant women in Ethiopia based on 2016 Ethiopian Demographic and Health Survey (EDHS) data.

## Methods

### Study design and setting

Secondary data analysis was conducted based on the 2016 Ethiopian Demographic and Health Survey (EDHS) data. EDHS 2016 was the fourth survey conducted in Ethiopia. It is the most populous country in the horn of Africa. The country covers 1.1 million square kilometers and has a great geographical diversity, which ranges from 4550 m above water level right down to the Afar depression to 110 m below the water level. There are nine regional states (list of regions in bracket) and two city administrations (Addis Ababa and Dire Dawa) subdivided into 68 zones, 817 districts, and 16,253 kebeles (lowest local administrative units of the country within the administrative structure of the country) [17].

### Data source, sampling procedure, and study participants

The study used data from the Ethiopian Demographic and Health Survey (EDHS) 2016. The EDHS 2016 used a two-stage stratified cluster sampling technique to select study participants using the 2007 Population and Housing Census (PHC) as a sampling frame. Stratification was achieved by separating each region into urban and rural areas. In total, 21 sampling strata have been created because the Addis Ababa region is entirely urban. In the first stage, a total of 645 Enumeration Areas (EAs) (202 in urban areas and 443 in rural areas) proportional to EA size were selected. At the second stage, 28 households per each cluster were systematically selected [17]. The study was conducted based on EDHS data by accessing from the DHS program official database www.measuredhs.com, after permission was secured through an online request by explaining the purpose of this study. The women’s Individual data set was used. The raw data was collected from all parts of the country among pregnant women using a structured and pre-tested questionnaire.

The surveys were based on a nationally representative probability sample that covered the entire country. Women aged 15–49 years were interviewed using a woman’s standard questionnaire and several variables like socio-economic and demographic information was collected from women and households.

The target participants of this study were all reproductive age women living in Ethiopia prior to 5 years of the survey and consequently, 15,683 reproductive age (15–49) women who were usual residents in the selected households before the survey were eligible and interviewed. Among those, women interviewed a weighted sample of 7589 pregnant mothers who had a pregnancy in the preceding 5 years were included in this study.

### Variables of the study

Iron supplementation use during pregnancy, coded as “1” if a pregnant woman was supplemented with iron and “0” if not supplemented with iron during pregnancy, was our outcome variable. After reviewing different kinds of literature, for our study, both individual and community-level variables were considered as independent/explanatory variables.

The individual-level variables include: maternal age (15–19, 20–24, 25–29, 30–34, 35–39, 40–44, and 45–49), religion, wealth index was assembled using household asset data via Principal Components Analysis (PCA) to categorize individuals into wealth quintile (poorest, poorer, medium, richer and richest), occupational status (employed and unemployed), family size, mass media exposure (exposure to mass media (indexed from television, newspaper, and radio), women’s education (categorized as no education, primary, secondary and higher, parity (categorized as primipara- women who are giving birth at the first time during the survey, multipara- women had more than one pregnancy resulting viable children), and Antenatal Care (ANC) visit (0–3 times -women didn’t have any ANC visit or less than 4 times ANC visiting and 4+ times- women had visit ANC at least 4 times in health institution).

The community-level variables were residence, region, perception of distance from the health facility, and community women education. Community-women education was created by aggregating the individual level factor women education since it was not directly found in the DHS data but known to a significant factor for the outcome variable. Since this variable was not normally distributed we used the median value to categorize it as low (communities with low proportions of women education) and high (communities with higher proportions of women education).

### Data management and analysis

After downloading the data from the DHS program official database (www.measuredhsprogram.com), editing, recording, and analysis were done using STATA 14, ArcGIS 10.6, and SaTscan version 9.6. The data were weighted using sampling weight, primary sampling unit, and strata to restore the representativeness of the data and to get a reliable estimate.

### Spatial analysis

For the spatial analysis, ArcGIS version 10.6 and SaTScan version 9.6 statistical software were used for exploring the spatial distribution, global spatial autocorrelation, spatial interpolation, and for identifying significant hotspot areas of iron supplementation. The Global spatial autocorrelation was done to assess whether the spatial distribution of iron supplementation is random or not. The spatial autocorrelation (Global Moran’s I) is the correlation coefficient for the relationship between a variable and its surrounding values. Moran’s I is a spatial statistics used to measure spatial autocorrelation by taking the entire data set and produce a single output. Moran’s I value ranges from-1 to 1. A value close to 1 shows a strong positive spatial autocorrelation whereas a value close to − 1 shows a strong negative spatial autocorrelation. If Moran’s I close to 0, it indicates that there is no spatial autocorrelation. A statistically significant Moran’s I value (*p* < 0.05) can lead to rejection of the null hypothesis (iron supplementation is randomly distributed) and indicates the presence of spatial dependence.

Getis-OrdGi* statistics were used to identify significant hotspot and cold spot areas of iron supplementation. Getis-OrdGi* statistics were computed to measure how spatial autocorrelation that varies over the study location and Z-score is computed to determine the statistical significance of clustering, and the *p*-value computed for the significance. Statistical output with high GI* indicates “hotspot” whereas low GI* means a “cold spot”. Kriging interpolation techniques were employed to predict the prevalence of iron supplementation among reproductive-age women based on the observed values. There are different types of interpolation techniques, for this study we choose ordinary Kriging interpolation techniques since it has a small root mean square error and residuals.

Spatial scan statistical analysis was done using SaTscan version 9.6 software to identify significant primary and secondary clusters. Bernoulli’s model was fitted since the outcome variable was binary. A woman who didn’t take iron supplementation was considered as cases whereas those who had taken iron supplementation were considered as control. The Bernoulli model requires data for cases, controls, and geographic coordinates. The default maximum spatial cluster size of < 50% of the population was used, as an upper limit, since it allowed both small and large clusters to be detected and ignored clusters that contained more than the maximum limit. The scanning window with maximum likelihood was the most likely performing cluster, and the *p*-value was assigned to each cluster using Monte Carlo hypothesis testing by comparing the rank of the maximum likelihood from the real data with the maximum likelihood from the random datasets. The primary and secondary bunches were identified and assigned *p*-values and ranked based on their likelihood ratio test, based on 999 Monte Carlo replications [18].

### Multi-level analysis

Due to the hierarchical nature of the DHS data, multilevel logistic regression analysis was done to identify the determinants of iron supplementation use. The Intra-class Correlation Coefficient (ICC) and Median Odds Ratio (MOR) were computed to measure the variation between clusters/to assess the community level variability, and Proportional Change in Variance (PCV) was calculated to assess how much iron supplementation use was explained by the final model [19, 20]. Four models were constructed for the multi-level logistic regression analysis. The first model was the null model without any explanatory variables, to calculate the extent of cluster variations on iron supplementation use among pregnant women. The second model was adjusted with individual-level variables, the third model was adjusted for community-level variables while the fourth was fitted with both individual and community level variables simultaneously. After comparing all models a model with low deviance was considered as a fitness model and the fourth model was the best-fitted model since it had lower deviance and higher likelihood.

In the multivariable multilevel logistic regression analysis variables with a *p*-value <, 0.05 were considered as statistically significant. Adjusted Odds Ratio (AOR) with their corresponding 95%CI was determined to identify factors associated with iron supplementation use among pregnant women. Multi-collinearity was also checked using the Variance Inflation Factor (VIF). Accordingly, all variables had VIF < 5 and tolerance greater than 0.1 indicating that there is no multi-collinearity.

## Results

### Socio-demographic characteristics of study participants

A total of 7589 respondents were included in the study. Above one fourth 2165, (28.5%) of the respondents were within the age range of 25–29 years. About 1654 (21.8%) respondents were from households with poorer wealth quantile category. The majority, 4406 (58%) of the respondents perceived distance from the health facility as a big problem whereas only 2414 (32%) had four or more ANC visits. Concerning media exposure, 2564 (33.8%) study participants had media exposure. Of all the respondents, 662 (87.2%) were rural dwellers. Regarding region, about 3129 (41.2%) were Oromia whereas only 33 (0.4%) were from Dire Dawa. Moreover, about 4799 (63.2%) of participants were from communities with lower community levels of female education (Table 1).

**Table 1:** Percentage distribution of characteristics of respondents in 2016 Ethiopian Demographic and Health Surveys

Characteristics	Weighted frequency	Percentage
**Age**		
15–19	339	4.5
20–24	1465	19.3
25–29	2165	28.5
30–34	1661	22
35–39	1206	16
40–44	546	7
45–49	207	2.7
**Residence**		
Rural	6620	87.2
Urban	969	12.8
**Religion**		
Orthodox	2882	38
Protestant	1651	21.8
Muslim	2824	37.2
Others^a^	232	3
**Marital status**		
Single	144	2
Married	7020	92.5
Widowed	95	1.2
Divorced	233	3
Separated	97	1.3
**Region**		
Somali	269	3.5
Tigray	537	7.1
Afar	71	1
Amhara	1632	21.5
Oromia	3129	41.2
Benishangul-Gumuz	81	1.1
SNNPR	1600	21.1
Gambelia	21	0.3
Harari	17.4	0.2
Addis Ababa	198.3	2.6
Dire Dawa	33.3	0.4
**Wealth index**		
Poorest	1651	21.7
Poorer	1654	21.8
Middle	1588	21
Richer	1427	18.8
Richest	1269	16.7
**Community women’s education**		
Lower community education	4799	63.2
Higher community education	2790	36.8
**Occupational status**		
Not working	4078	53.7
Working	3511	46.3
**perception of the distance to health facility**		
A big problem	4406	58
Not a big problem	3183	42
**Parity**		
0–4	4624	61
5–9	2732	36
10+	233	3
**Family size**		
1–4	2331	31
5–9	4875	64
10+	383	5
**ANC visit**		
0–3 times	5175	68
4 + times	2414	32
**Media exposure**		
Not have media exposure	5025	66.2
Have media exposure	2564	33.8

^a^key: others- traditional, catholic

### Regional prevalence of iron supplementation among reproductive-age women

**Fig. 1:**
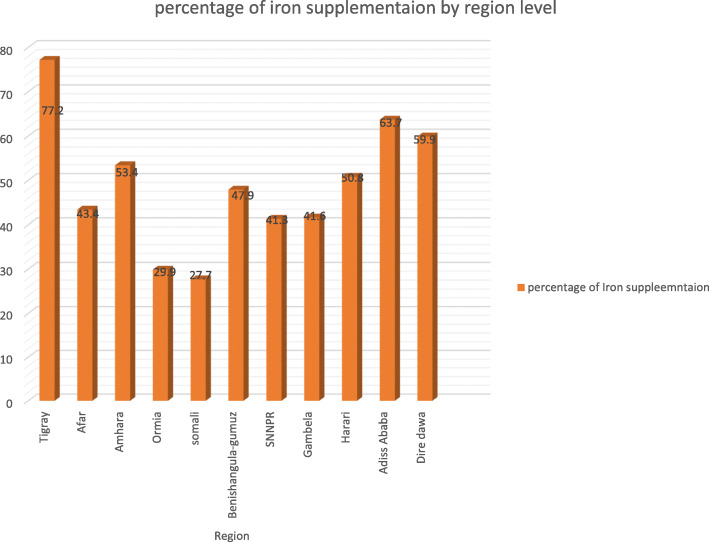
Regional prevalence of iron supplementation among pregnant women in Ethiopia, 2016

The prevalence of iron supplementation during pregnancy varies across the country. The highest and lowest prevalence of iron supplementation during pregnancy was observed in Tigray (77.2%) and Somali (27.7%) regions respectively (Fig. 1).

### Spatial analysis of Iron supplementation

**Fig. 2:**
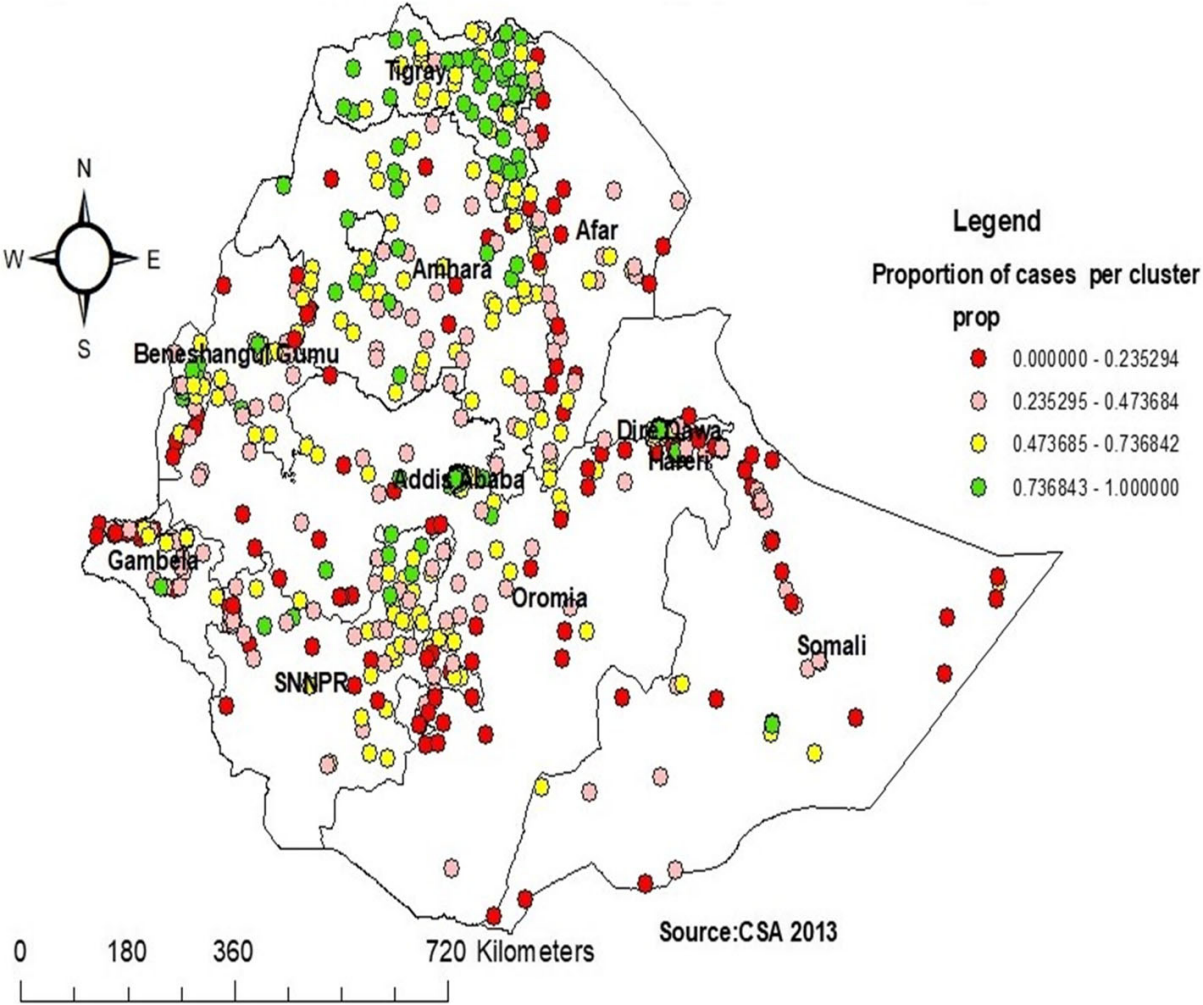
Spatial distribution of Iron supplementation among pregnant women in Ethiopia, 2016

**Fig. 3:**
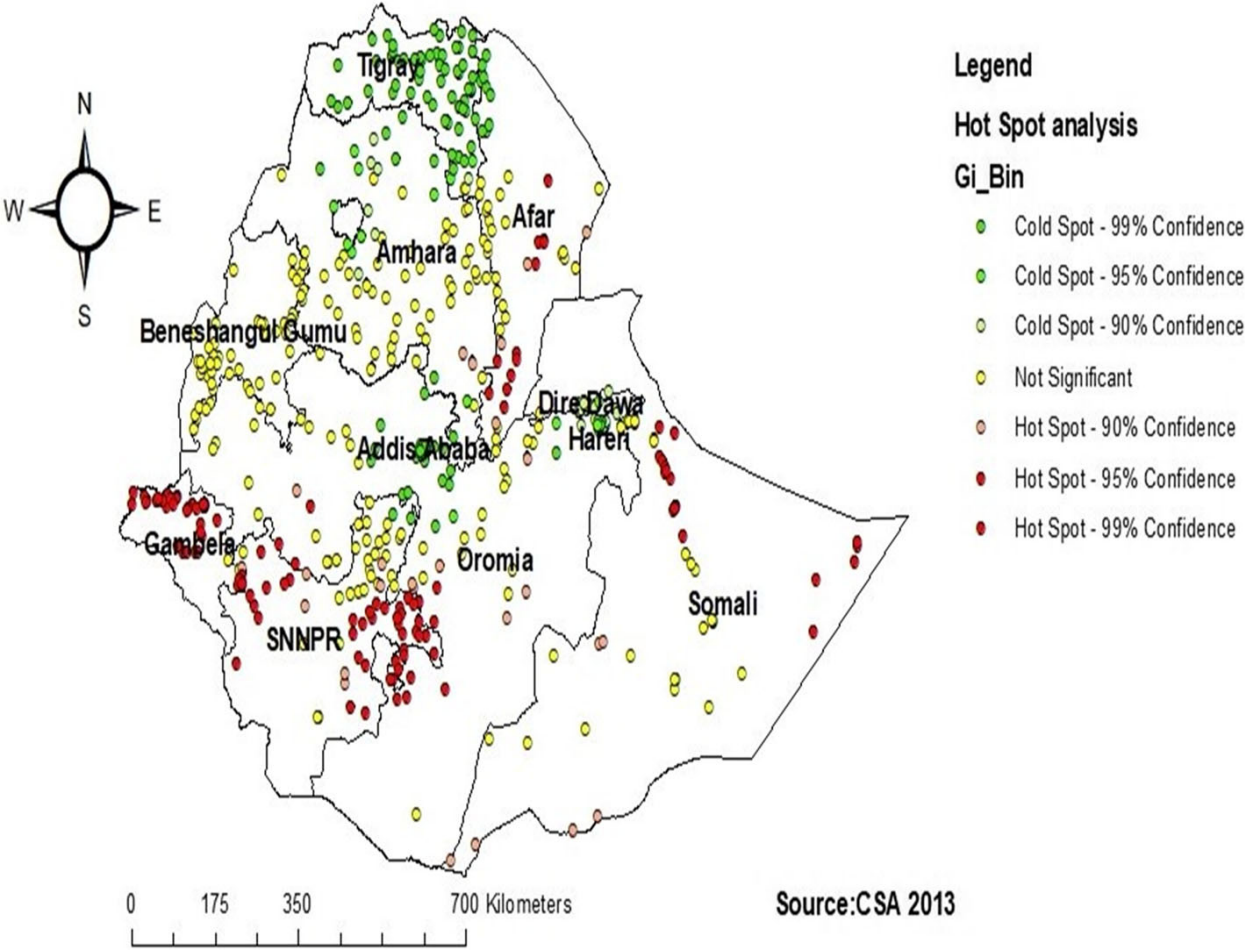
Hot spot and cold spot identifications of Iron supplementation among pregnant women in Ethiopia, 2016

The spatial distribution of iron supplementation was non-random in Ethiopia with Global Moran’s I value of 0.30 (*p* < 0.001) (Fig. 3). The high prevalence of iron supplementation was located in the Tigray, Northeast Amhara, Beneshangul Gumuz, Addis Ababa, and Northeast SNNPR regions. Whereas areas with a low prevalence of iron supplementations were identified in Somali, Afar, Southwest Oromia, West Gambella, and Southwest part of Addis Ababa (Fig. 2).

In the Getis OrdGi statistical analysis, significant hotspot areas (areas where iron supplementations were poor) were located in the Northeast Somali, South Afar, North West Gambela, West, and east SNNPR, and Southwest Oromia regions (*P*-value< 0.01). Whereas the significant cold spot areas (areas with high iron supplementation) were located in Tigray, North Amhara, East Addis Ababa, and North West Harari regions (Fig. 3).

**Fig. 4:**
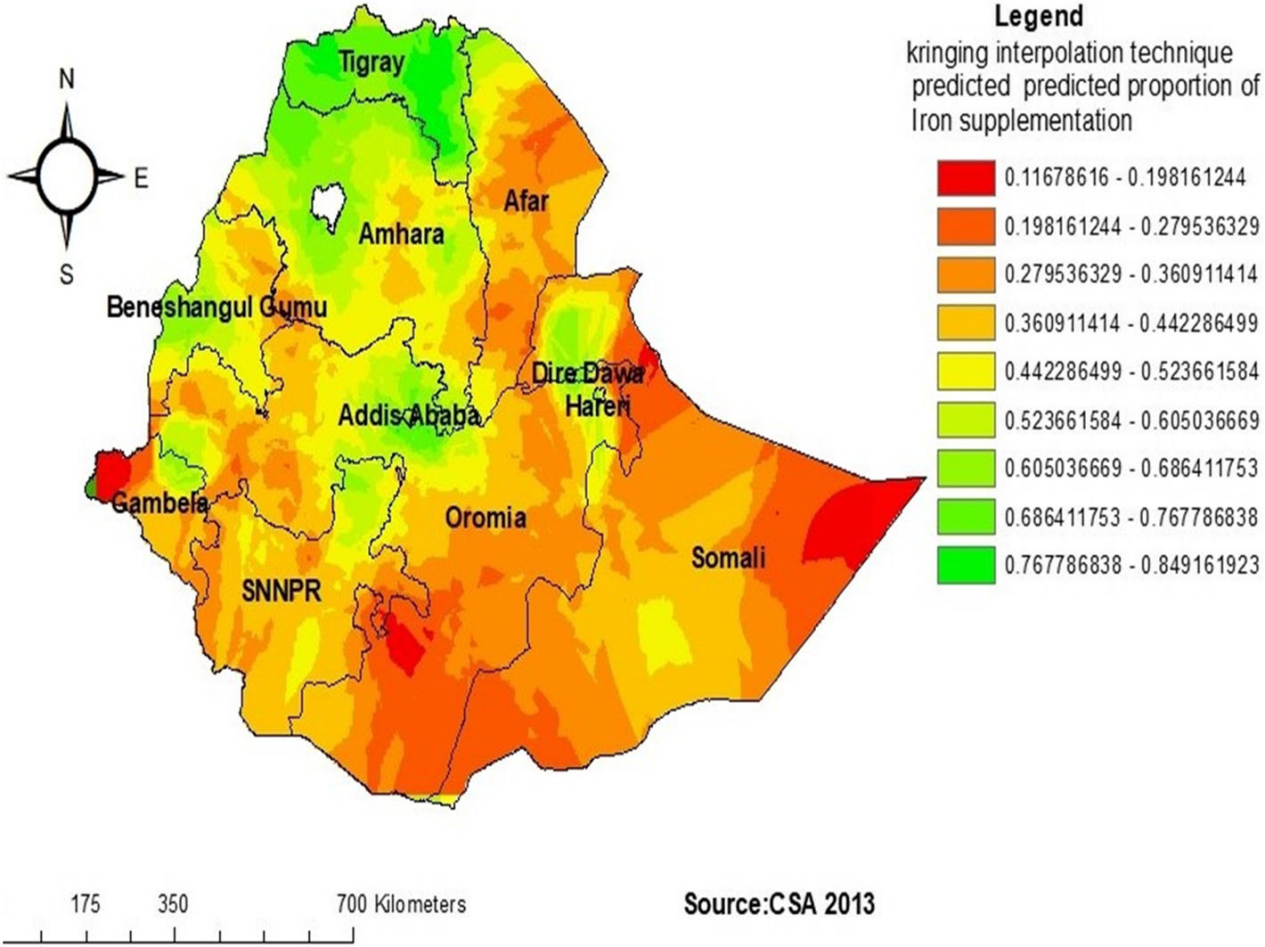
Kriging interpolation of iron supplementation among pregnant women in Ethiopia, 2016

In the Kriging interpolation; the predicted low prevalence of iron supplementations were identified in the Northwest Gambela, east Somali, southwest Somali, North West Oromia, and Northeast Afar regions whereas, the predicted high prevalence of iron supplementation were identified in the Tigray, Northwest Amhara, Northeast Addis Ababa, West Beneshangul Gumuz, Northeast Addis Ababa, and North SNNPR regions (Fig. 4).

**Fig. 5:**
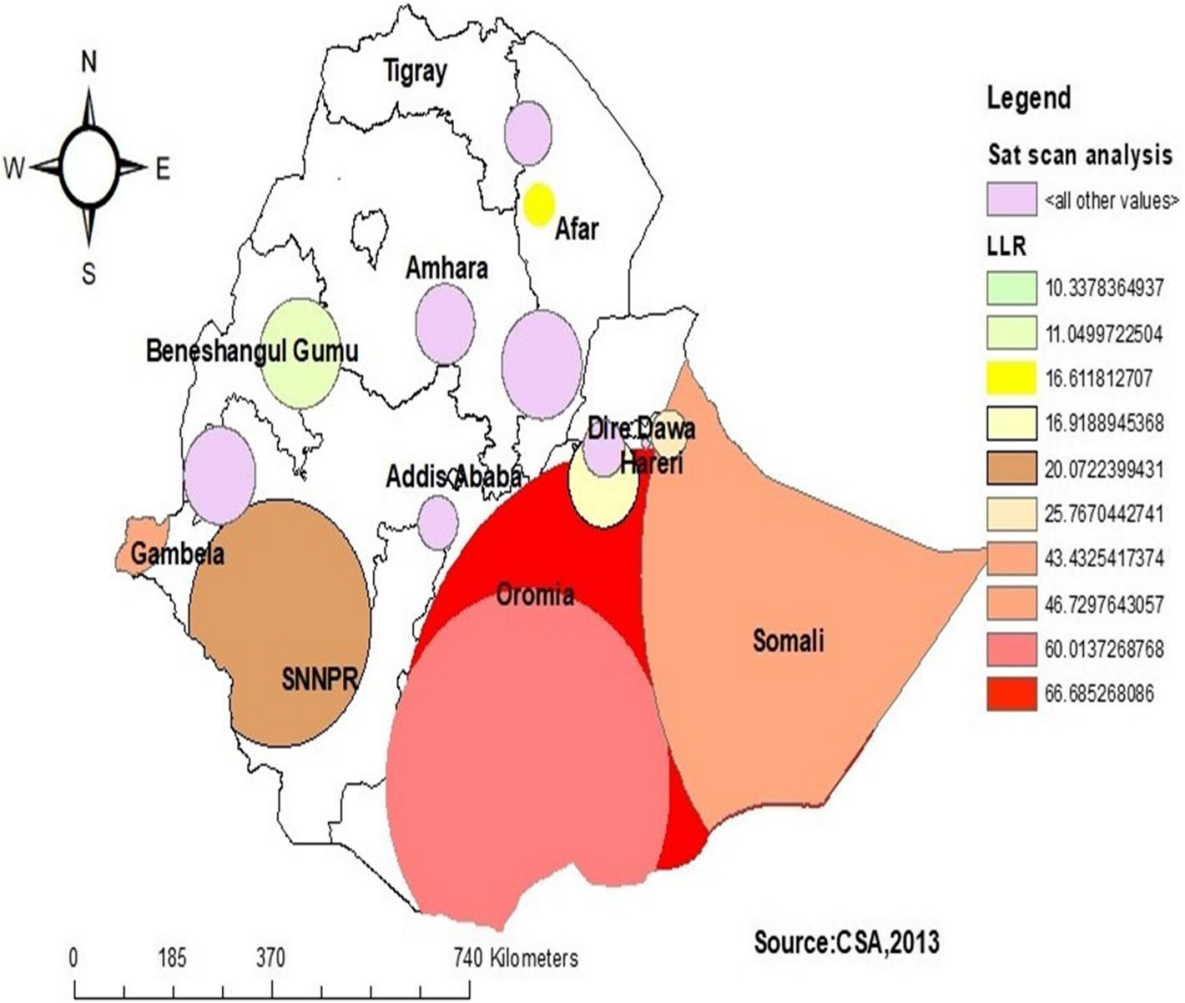
Spatial Sat Scan analysis of iron supplementation among pregnant women in Ethiopia, 2016

In the spatial scan statistics, a total of 271 significant clusters were identified, of these 89 were primary (most likely) clusters. The primary clusters were located in southwest Somali and central parts of the Oromia region centered at 5.330795 N, 41.837597 E of geographic location with 441.87 km radius, a Relative Risk (RR) of 1.35 and Log-Likelihood ratio (LLR) of 66.68, at *p* < 0.001. It showed that women within the spatial window had a 1.35 times higher likelihood of low iron supplementation than women outside the spatial window (Fig. 5) (Table 2).

**Table 2:** Sat Scan analysis of iron supplementation among pregnant women in Ethiopia, 2016

Cluster	Enumeration area (cluster)identified	Coordinate/radius	Population	Case	RR	LLR	*p*-value
1 (89)	556, 394, 480, 187, 520, 318, 278, 208, 164, 358, 377, 85, 289, 286, 472, 138, 452, 7, 492, 422, 543, 92, 490, 198, 171, 95, 34, 146, 82, 497, 518, 123, 405, 562, 521, 588, 553, 26, 468, 316, 458, 601, 213, 398, 319, 576, 313, 619, 529, 365, 600, 21, 245, 445, 232, 589, 12, 214, 372, 634, 251, 32, 182, 573, 476, 391, 574, 524, 239, 122, 308,216, 578, 215, 116, 22, 408, 148, 438, 522, 412, 513, 454, 506, 580, 68, 115, 133, 501, 453, 607, 568	(5.330795 N, 41.837597 E) / 441.87 km	1240	852	1.35	66.68	< 0.001
2 (37)	377, 394, 422, 7, 34, 289, 480, 398, 316, 601, 82, 556, 405, 21, 518, 468, 232, 472, 600, 208, 313, 182, 445, 574, 32, 576, 286, 634, 26,365, 452, 520, 12, 215, 216, 408	**(**5.203234 N, 40.019732 E) / 261.38 km	510	391	1.47	60.0	< 0.001
3 (12)	66, 618, 309, 435, 536, 370, 507, 592, 104, 260, 233, 69	(8.389747 N, 33.258557 E) / 71.61 km	146	132	1.7	46.72	< 0.001
4 (49)	630, 378, 269, 629, 77, 146, 92, 490, 543, 171, 492, 198, 95, 497, 458, 588, 553, 521, 138, 214, 33, 573, 251, 239, 116, 85, 358, 22,164, 527, 568, 277, 439, 64, 57, 278, 210, 8, 186, 566, 1, 318, 622,436, 212, 454, 501	7.717178 N, 46.991580 E) / 555.85 km	587	423	1.37	43.43	< 0.001
5 (10)	1, 566, 622, 186, 307, 436, 212, 8, 210, 419	**(**9.505470 N, 42.438628 E) / 33.79 km	142	117	1.54	25.76	< 0.001
6 (42)	477, 325, 207, 437, 376, 154, 168, 177, 552, 459, 371, 243, 465, 299, 526, 554, 197, 46, 586, 489, 119, 338, 76, 326, 555, 470, 337, 432,486, 447, 448, 62, 306, 227, 446, 411, 219, 558, 270, 593, 265, 406	(7.173968 N, 35.802680 E) / 170.61 km	509	343	1.27	20.07	< 0.001
7 (7)	372, 93, 412, 333, 476, 506, 453	**(**8.949350 N, 41.312402 E) / 65.76 km	105	85	1.51	16.91	< 0.001
8 (2)	544, 599	(12.349981 N, 40.242399 E) / 29.62 km	34	33	1.8	16.61	< 0.001
9 (16)	150, 36, 183, 559, 184, 246, 533, 244, 137, 364, 35, 498, 615, 320, 515, 494	(10.512406 N, 36.129050 E) / 76.33 km	198	139	1.31	11.04	0.011
10(7)	20, 276, 283, 547, 102, 37, 55	(10.381987 N, 40.265796 E) / 75.25 km	91	70	1.43	10.33	0.019

### Multilevel logistic regression analysis for assessing factors associated with iron supplementation among reproductive-age women

#### Random effect analysis results

The intraclass correlation within the null model indicated that 27% of the variability in iron supplementation was attributable to community-level variability. Also, the median odds ratio which was 3.3 in the null model indicates that there was a variation of iron supplementation between clusters. If we randomly select women from two different clusters women at the cluster with higher iron supplementation had 3.3 times higher odds of experiencing iron supplementation as compared with women at the cluster with lower iron supplementation. Moreover, as shown by PCV in the final model, about 70% of the variability in iron supplementation was explained by both individual and community-level factors (Table 3).

**Table 3:** Multivariable multilevel logistic regression analysis result of both individual and community-level factors associated with iron supplementation among pregnant women in Ethiopia, EDHS 2016

Variables	Null model	Model II AOR(95%CI)	Model III AOR(95%CI)	Model IV AOR(95%CI)
Age				
15–19		1.09 [0.67–1.75]		1.19 [0.74–1.91]
20–24		1.17 [0.78–1.76]		1.22 [0.82–1.84]
25–29		1.10 [0.75–1.63]		1.17 [0.79–1.72]
30–34		0.96 [0.65–1.42]		1.00 [0.68–1.48]
35–39		0.86 [0.58–1.28]		0.86 [0.58–1.28]
40–44		0.69 [0.45–1.07]		0.68 [0.44–1.05]
45–49		1		1
Education				
No education		1		1
Primary		1.26[1.09–1.46]		1.24 [1.07–1.44]**
Secondary		1.29[1.01–1.65]		1.27 [0.99–1.63]
Higher		1.56[1.12–2.18]		1.59 [1.14–2.21]**
Residence				
Rural			1	
Urban			1.81 [1.42–2.33]	1.20 [0.89–1.61]
Region				
Somali			1	1
Tigray			9.18 [6.35–13.28]	5.29 [3.68–7.59]**
Afar			1.75 [1.22–2.51]	1.61 [1.14–2.28]**
Amhara			3.08 [2.19–4.33]	2.04 [1.45–2.87]**
Oromia			1.25 [0.89–1.75]	0.80 [0.57–1.12]
Benishangul-Gumuz			2.37 [1.62–3.46]	1.35 [0.93–1.96]
SNNPR			1.91 [1.35–2.71]	1.10 [0.78–1.55].
Gambelia			1.02 [0.68–1.52]	0.72 [0.49–1.06]
Harari			2.10 [1.39–3.16]	1.44 [0.97–2.16]
Addis Ababa			1.92 [1.24–2.96]	0.83 [0.54–1.28]
Dire dawa			2.95 [1.94–4.48]	1.52 [1.02–2.29]*
Wealth index				
Poorest		1		1
Poorer		1.42 [1.18–170]		1..37 [1.14–1.68]**
Middle		1.36 [1.11–1.66]		1.30 [1.07–1.63]**
Richer		1.41 [1.14–1.75]		1.37 [1.10–1.70]**
Richest		1.40 [1.11–1.77]		1.12 [0.84–1.49]
Community women’s education				
Lower community education			1	1
Higher community education			1.61 [1.32–1.97]	1.21 [0.99–1.48]
Occupational status				
Unemployed		1		1
Employed		1.18 [1.04–1.33]		1.10 [0.97–1.25]
Perception of the distance to a health facility				
A big problem			1	1
Not a big problem			1.43 [1.26–1.61]	1.31 [1.15–1.49]**
Parity				
primipara		1		1
multipara		0.99 [0.83–1.19]		1.02 [0.86–1.22]
Family size				
1–4		1		1
5–9		1.00 [0.87–1.15]		1.03 [0.89–1.18]
10+		1.01 [0.77–1.35]		1.10 [0.84–1.44]
ANC visit				
0–3 times		1		1
4 + times		3.79 [3.32–4.31]		3.59 [3.15–4.09]**
Media exposure				
Not have exposure		1		1
Have exposure		1.34 [1.15–1.55]		1.28 [1.11–1.49]**
**Constant**	0.88 [0.80–98]	0.29 [0.19–0.46]	0.24 [0.18–0.31]	0.17 [0.11–0.29]
Model comparison and random effects				
ICC	0.27 [0.24–31]			
Log likelihood (LL)	− 4552.55	− 4206.89	− 4373.57	− 4107.52
Deviance	9105.1	8413.78	8747.14	8215.04
PCV	Ref	0.46	0.63	0.70
MOR	3.3(2.7,4.5)	2.03(1.78,2.39)	1.62(1.46,1.85)	1.47(1.34,1.65)

Key: *AOR* Adjusted odds ratio, *CI* confidence interval, *ICC* intra-cluster correlation, *MOR* median odds ratio; 1: reference group; *p*-value 0.05–0.01 *: *p*-value < 0.01 **: *ANC* antenatal care visit

#### The fixed effect analysis result

Of the four fitted models, the final multilevel logistic regression model (model 4) was the best-fitted model for this study because this model had a high likelihood and low deviance.

In the multivariable multilevel logistic analysis, individual-level factors like wealth index, ANC visit, women’s education, and media exposure were found to be significantly related to the odds of iron supplementation. Among the community-level factors region, and perception of distance from the health facility were significantly related to iron supplementation.

Women who attended a minimum of four ANC visits had 3.59 times (AOR = 3.59, 95%CI: 3.15, 417) higher odds iron supplementation use as compared to those they didn’t attend the minimum requirement of ANC visit (< 4 ANC visit). The odds of iron supplementation were 1.28 times (AOR = 1.18, 95%CI: 1.11, 1.49) higher among those mothers who had media exposure as compared to their counterparts. Those mothers who were from the poorer, middle, and richer households had 1.37 (AOR = 1.37, 95%CI: 1.14, 1.65), 1.30 (AOR = 1.30, 95%CI: 1.07, 1.60), and 1.37 (AOR = 1.37, 95%CI: 1.10, 1.70) times higher odds of iron supplementation as compared with women from poorest households. Regarding the perception of distance from the health facility, women who perceived distance from the health facility had 1.31 (AOR = 1.31, 95%CI: 1.15, 1.49) times higher odds of iron supplementation as compared to their counterparts.

With adjusting other covariates, women’s in Tigray, Afar, Amhara, and Dire Dawa regions were 5.29 (AOR = 5.29, 95%CI: 3.68, 7.59), 1.61 (AOR = 1.61, 95%CI: 1.14, 2.28), 2.04 (AOR = 2.04, 95%CI: 1.45, 2.87) and 1.52 (AOR = 1.52, 95%CI: 1.02, 2.29) times higher iron supplementation use than that of women’s in Somali region, respectively.

Moreover, women with primary and higher education were 1.24 [AOR = 1.07, 95%CI, 1.07, 1.44), and 1.59 (AOR = 1.59=, 95%CI: 1.14, 221) times higher odds of iron supplementation as compared to those women with no education, respectively (Table 3).

## Discussion

The spatial analysis revealed that the spatial distribution of iron supplementation among reproductive-age women was significantly varied across the country. The significant hotspot areas with low iron supplementation were located in Northeast Somali, South Afar, North West Gambela, West and East a part of SNNPR, and Southwest Oromia regional states were statistically significant hot spot areas for iron supplementation (low iron supplementation) and the SaTscan analysis identifies significant primary (Most likely clusters) clusters in Southwest Somali and central part of Oromia region. The possible explanation might be due to the lowest ANC utilization rate was reported in hot spot areas as compared to cold spot areas lowest ANC service utilization in the border areas of the country [17]. This might be attributed to the discrepancy within the distribution of maternal health service, and inaccessibility of infrastructure within the border areas of Somali, and Benishangul regions [21]. Moreover, these regions also are relatively rural residents and that they couldn’t access health facilities and ladies might not have awareness about the iron supplementation program, and its benefit during their pregnancy might not have good access to maternal health care services. In several studies also evidence showed that in Somali,Hareri, and Afar regions the prevalence of anemia among reproductive-age women was high [22, 23].

ANC visits, community women’s education, region, media exposure, household wealth index, and perception distance to health facility were associated factors of iron supplementation use among pregnant women in Ethiopia.

ANC visits were significant determinants of iron supplementation. This was consistent with the study findings reported in the study is supported by a study done in Ethiopia [13], Tanzania [24], Senegal [15], Pakistan [25], and India [14]. The possible justification might be mothers who had adequate ANC visits (four and above) may have information about the importance of iron supplementation and should have a positive attitude towards maternal health services including iron use compared to their counterparts. In this study, women’s education is one of the important factors for iron supplementation among pregnant women. The chance of getting an iron tablet higher among women with primary and higher education as compared to no education. The finding of this study is similar to the finding of studies in India [14], Pakistan [25], and Senegal [26]. This could be due to education is a vital tool to enhance knowledge of pregnant women about the consequence of iron deficiency and show the ways to handle these deficiencies. This means educated pregnant women have an excellent ability to take maternal health services like iron supplementation during pregnancy [27–29].

Media exposure is positively associated with iron supplementation among pregnant women. This study was supported by a study in Asia [30]. This is because different maternal health services including the importance of taking iron during pregnancy are frequently given to the community through mass media. Therefore, pregnant women who are exposed to media would have a far better understanding of the advantages of taking iron tablets compared to those who do not have media exposure.

The region is also significantly associated with iron supplementation among pregnant women. Those pregnant women from Tigray, Afar, Amhara, and Dire Dawa regions had a higher chance of taking the iron tablet as compared to the Somali region. This could be explained by the difference in the coverage of ANC utilization across these regions, lower ANC visit was observed in the Somali region compared to other regions [17]. This is often the very fact that ANC visits are the major route to deliver iron supplementation for pregnant women in Ethiopia. That’s why pregnant mothers in Tigray, Afar, Amhara, and Dire Dawa regions had better iron supplementation than the Somali region.

The household wealth index is another important factor of iron supplementation among pregnant women. Mothers who were in poorer, middle, and richer wealth quantile categories higher chance of taking iron supplementation as compared within the poorest wealth quantile. This study finding is consistent with a study finding reported in Senegal [26]. The possible explanation could be due to the reason that the richest women have good access to maternal health care service utilization [31]. In this study, the distance to a health facility is significantly associated with iron supplementation. The odds of iron supplementation among women where the distance to the health facility was not a big problem were higher as compared to women who had a big problem to reach a health facility. This might be due to the reason that women who can easily access health care could have a higher likelihood of utilizing maternal health care services like ANC visits.

Ethiopia has performed amazingly to decrease maternal and child health burden. Particularly, strong strategic leadership and political guarantee to the creation and delivery of health programs that drive primary care are especially significant for maternal and child health care. Anemia among pregnant women had a double burden and it is a very serious problem. To prevent this problem administering iron tablets for pregnant women is a very critical issue to create a healthy community. In Ethiopia, the coverage of iron supplementation during pregnancy is still low and has not fulfilled the World Health Organization (WHO) standard recommendations. Besides, iron supplementation among pregnant women varies across Ethiopia. Therefore, investing more in the determinant factors and making an intervention on the high-risk areas by designing different policies and strategies very crucial issue to improve maternal and child health in Ethiopia.

### Strength and limitation of the study

The strengths of this study, first, the study was based on nationally representative weighted data and can be generalized at the national level. Second, the use of spatial analysis to explore the spatial distribution and significant hotspot areas of iron supplementations and the use of multilevel analysis. The limitations of this study were, it shares the limitations of cross-sectional studies. Besides, self-reported data were included in this study like the perception of the distance to health facility lead to self-reported bias.

## Conclusion

This study investigated that the spatial clustering of poor iron supplementation among pregnant women was found in North East Somali, South Afar, North West Gambela, Western, and Eastern parts of SNNPR and Southwest Oromia regions. Community education, household wealth index, ANC visit, region, media exposure, and perception of distance to a health facility were significant predictors of iron supplementation among pregnant women. To enhance iron supplementation, targeted intervention should be taken on these identified high-risk areas. Intervention programs that can increase the frequency of ANC visits, education level, and use of mass media need to be done.

## Data Availability

All relevant data are included in the manuscript. It also possible to access the data set online from the DHS program.

## Abbreviations

AIC: Akaike information criteria
ANC: Antenatal Care
AOR: Adjusted Odds Ratio
BIC: Bayesian information criteria
COR: Crude Odds Ratio
EDHS: Ethiopian Demographic health survey
ICC: Intra-Class Correlation
MOR: Median Odds Ratio
PCV: Proportional Change in Variance
