# Correction to: Spatial distribution and determinants of iron supplementation among pregnant women in Ethiopia: a spatial and multilevel analysis

**DOI:** 10.1186/s13690-021-00675-4

**Published:** 2021-08-24

**Authors:** Chilot Desta Agegnehu, Getayeneh Antehunegn Tesema, Achamyeleh Birhanu Teshale, Adugnaw Zeleke Alem, Yigizie Yeshaw, Sewnet Adem Kebede, Alemneh Mekuriaw Liyew

**Affiliations:** 1 School of Nursing, College of Medicine and Health Sciences and Comprehensive Specialized Hospital, University of Gondar, Gondar, Ethiopia; 2 Department of Epidemiology and Biostatistics, Institute of Public Health, College of Medicine and Health Sciences, University of Gondar, Gondar, Ethiopia; 3 Department of Physiology, School of Medicine, College of Medicine and Health Sciences, University of Gondar, Gondar, Ethiopia

**Correction to: Arch Public Health 79, 143 (2021)**

**https://doi.org/10.1186/s13690-021-00669-2**

Following publication of the original article [1], the author noticed that the first author’s family name was incorrectly spelled as “Agegenehu”.

The correct spelling of the family name “Agegnehu” has been provided in this Correction.

The original article [1] has been updated.
